# ﻿Three new taxa of lichen genus *Trimmatothelopsis* (Acarosporales, Acarosporaceae) from China

**DOI:** 10.3897/mycokeys.120.158033

**Published:** 2025-08-13

**Authors:** Jiaxin Wang, Fuhui Liang, Zuntian Zhao, Ling Hu

**Affiliations:** 1 College of Geography and Environment, Shandong Normal University, Jinan 250300, China Shandong Normal University Jinan China; 2 Key Laboratory of Plant Stress Research, College of Life Sciences, Shandong Normal University, Jinan 250300, China Shandong Normal University Jinan China

**Keywords:** Ascus stains, new species, saxicolous lichen, taxonomy

## Abstract

Three new species *Trimmatothelopsisanthracina*, *T.knudsenii*, and *T.shandongensis* are reported from the eastern coast of China, based on morphological, chemical and molecular data (ITS, LSU, and mtSSU). All three new species have IKI+ blue ascus. *Trimmatothelopsisanthracina* is characterized by carbonized apothecia, a biofilm at the immature apothecia base, and IKI+ blue turning red hymenial gel. *Trimmatothelopsisknudsenii* is characterized by rimose thallus, numerous reddish brown apothecia, and IKI+ blue turning red hymenial gel. *Trimmatothelopsisshandongensis* is characterized by dull brown thallus, thick hymenium, IKI+ red hymenial gel, and small asci. Comprehensive descriptions, detailed illustrations, and phylogenetic analysis of the new taxa are presented. Additionally, a worldwide key to *Trimmatothelopsis* species, including 21 species, is provided.

## ﻿Introduction

The genus *Trimmatothelopsis* (Acarosporaceae) was first established by Zschacke (1934), with *T.versipellis* as the type species. The monophyletic genus *Trimmatothelopsis* is characterized by globose apothecia with the disc usually less than 0.5 mm in diam, the hymenium usually 150–350 μm high, two types of ascus stain (*Acarospora*-type with IKI- tholus or IKI+ blue tholus), long conidia and no secondary metabolites (Knudsen and Lendemer 2016; Knudsen et al. 2023a). Before this study, the genus included 18 reported species, occurring on calcareous or non-calcareous rock or on soil, distributed across Asia, Australasia, Europe, and North America (Zschacke 1934; Knudsen and Lendemer 2016; Knudsen et al. 2021a, 2022, 2023a; Park et al. 2023). For a history of the genus and its nomenclature see Knudsen et al. 2021a.

The main form of thallus in *Trimmatothelopsis* is areoles or squamules, dispersed and/or with congregations of thalline units. *Trimmatothelopsisamericana*, for instance, differs in having carbonized lecideine apothecia with algal layer occurring in a biofilm at its base. In this genus, all species have globose hymenium which are widest at the equator, with hymenium heights typically exceeding 200 μm high. However, three species exhibit lower hymenia, *T.californica* is 110–170 μm high, *T.coreana* is up to 190 μm high, and *T.ireneana* is 130–175 μm high. The asci in *Trimmatothelopsis* are variable. Seven species have an IKI+ blue ascus, *T.americana*, *T.gordensis*, *T.mexicana*, *T.montana*, *T.novomexicana*, *T.serpentinicola*, and *T.wirthii*.

Conidia were reported from 13 of 18 species in *Trimmatothelopsis* (*T.americana*, *T.benedarensis*, *T.californica*, *T.gordensis*, *T.mexicana*, *T.novomexicana*, *T.oreophila*, *T.rhizobola*, *T.schorica*, *T.terricola*, *T.versipellis*, *T.wendyana*, *T.wirthii*) (Knudsen et al. 2011, 2023a, 2024; Park et al. 2023). The conidia showed similar size in most species, which were 3–6 × 1–1.5 μm. However, three species exhibited significant differences, *T.californica* produced the smallest conidia (1.8–2.8 × 0.8–1.3 μm), while *T.mexicana* produced the longest conidia (up to 13.9 × 1.5 μm), and *T.wirthii* produced the second longest conidia (up to 11 × 1.5 μm). In the family Acarosporaceae, the long conidia distinguish *Trimmatothelopsis* from the other genera, except in the monotypic genus *Lithoglypha* which also produces long conidia 3.5–7.5 × 0.8 μm. Unfortunately, no sequences of *Lithoglypha* are available so far. In these 13 species, the conidiogenous cells were also long, which were 5–20(–40) × 1–3 μm, except in *T.benedarensis*, *T.oreophila*, and *T.wendyana*.

Before this study, two *Trimmatothelopsis* species had been reported from China. *Acarosporadispersa*, as a synonym of *T.dispersa*, was reported from the east Tianshan Mountains, Xinjiang Uygur Autonomous Region. The specimen was described with a low hymenium (80–115 μm), small asci (45–54 × 11–11.5 μm), and non-globose apothecia (as shown in Plate 1 in the original paper) (Burabiya et al. 2015). However, *T.dispersa* was described with a higher hymenium (150–200 μm), larger asci (100 × 10–25 μm), and globose apothecia (Knudsen and Lendemer 2016; Knudsen et al. 2023a). Due to these different morphological features, this specimen significantly does not belong to the genus *Trimmatothelopsis*. Another species, *T.versipellis* was reported from Kunyu Mountain Nature Reserve. After redetermination of the specimen, this species should be *T.anthracina* (see discussion below).

During the surveys of lichen on the eastern coast of China, three interesting species were collected from the non-calcareous rock. Based on morphological, chemical, and phylogenetic analyses, we found that they were different from all known species in *Trimmatothelopsis*. Here, we describe three new species within the genus *Trimmatothelopsis*.

## ﻿Materials and methods

### ﻿Morphological and chemical analyses

The specimens were collected from the Shandong and Fujian Provinces and preserved in the Lichen Section of Botanical Herbarium, Shandong Normal University, Jinan, China (SDNU). Morphological characters were observed using a dissecting microscope (COIC XTL7045B2) and photographed using a microscope (Olympus SZX16) with a DP72 camera system. The anatomical characters were observed and measured using a polarizing compound microscope (Olympus CX41) and photographed using a microscope (Olympus BX61) with a DP72 camera system. Distilled water was used as a mounting slide solution. The amyloid reaction of the hymenial gel and subhymenium was tested with fresh undiluted IKI (Lugol’s iodine solution, 1%) (Knudsen and Kocourková 2018). The ascus stain was studied in IKI (Hafellner 1993). The lichen secondary metabolites were analyzed and identified by thin-layer chromatography (TLC) using solvent C (Orange et al. 2010).

### ﻿DNA extraction, PCR amplification and sequencing

The genomic DNA was extracted from the lichen thalli with apothecia using the Sigma-Aldrich REDExtract-N-Amp Plant PCR Kit (St. Louis, MO, USA) following the manufacturer’s protocol. The ITS, LSU, and mtSSU regions were respectively amplified using the primer pair ITS1F/ITS4 (White et al. 1990; Gardes and Bruns 1993), LR0R/LR5 (Vilgalys and Hester 1990; Rehner and Samuels 1995) and mrSSU1/mrSSU3R (Zoller et al. 1999). PCR reactions were carried out in 25 μL reaction system containing 1 μL of genomic DNA, 1 μL of each primer, 12.5 μL of 2 × Taq PCR MasterMix (Tiangen, Beijing, China), and 9.5 of μL dd H_2_O. Conditions for the ITS: initial denaturation 95 °C for 5 min, followed by five cycles (95 °C for 33 s, 56 °C for 30 s, and 72 °C for 30 s), then ten cycles (95 °C for 30 s, 54 °C for 30 s, and 72 °C for 30 s), and twenty cycles (95 °C for 30 s, 50 °C for 30 s, and 72 °C for 30 s) with a final extension 72 °C for 15 min (Knudsen et al. 2025). Conditions for the LSU: initial denaturation 98 °C for 3 min, followed by thirty-five cycles (98 °C for 10 s, 56 °C for 10 s, and 72 °C for 15 s) with a final extension 72 °C for 5 min (Dou et al. 2024). Conditions for the mtSSU: initial denaturation 94 °C for 3 min, followed by thirty-five cycles (94 °C for 15 s, 52 °C for 30 s, and 72 °C for 90 s) with a final extension 72 °C for 10 min (Zhang et al. 2024). Polymerase chain reaction (PCR) products were sequenced by the Sangon Biotech company (Jinan, China).

### ﻿Sequence alignment and phylogenetic analysis

A BLAST search was carried out to identify similar sequences in GenBank (http://www.ncbi.nlm.nih.gov/BLAST/). BLAST analysis of three genes (ITS, LSU, and mtSSU) from specimens of three new species indicated that they belong to *Trimmatothelopsis*, Acarosporaceae. To confirm its phylogenetic placement, the sequences from this genus and related genera in the Acarosporaceae were downloaded from GenBank. The species *Pycnorasorophora* was used as the outgroup. Geneious Prime was used to assemble and edit the sequences and generated a single matrix for ITS, LSU, and mtSSU. Each matrix was aligned using an online version of MAFFT v. 7 (https://mafft.cbrc.jp/alignment/server/). Geneious Prime was used to concatenate the ITS, LSU, and mtSSU genes and produce a three-locus dataset. The concatenated data matrix comprised 2268 characters (612 for ITS, 887 for LSU, and 769 for mtSSU).

**Figure 1.**
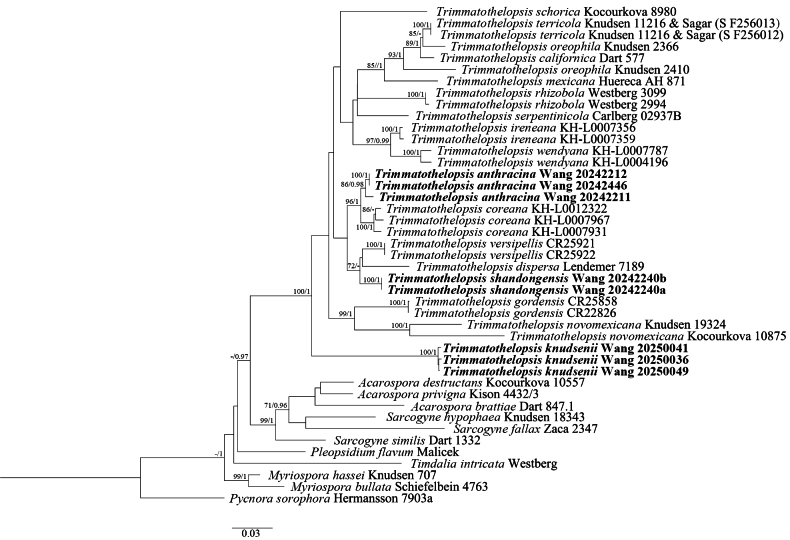
Phylogenetic tree generated from Maximum Likelihood (ML) analysis, based on a combined dataset of ITS, LSU, and mtSSU. Bootstrap support values of Maximum Likelihood (BS) ≥ 70%, and Bayesian posterior probabilities (PP) ≥ 0.95 are given near the nodes as BS/PP. Newly described species are marked in bold. Scale = 0.03 substitution per site.

Phylogenetic relationships were inferred using Maximum Likelihood (ML) (Fig. 1) and Bayesian inference (BI) (Suppl. material 1). The ML analysis was performed by RAxML v. 8.2.12 (Stamatakis 2014), using 1000 bootstrap replicates and a GTRGAMMA model on the CIPRES Web Portal (http://www.phylo.org) (Miller et al. 2010). For BI analysis, PartitionFinder 2 was used to determine the best-fit model for each partition (Lanfear et al. 2016). Based on the results, we used the GTR+I+G for ITS, LSU, and mtSSU. The BI analysis was performed using MrBayes v. 3.2.7 with 2 independent analysis runs for 1 million generations. Each run included four chains, parameters were sampled every 1000 generations and 25% were discarded as burn-in (Ronquist et al. 2012). ML bootstrap values (BS) ≥ 70% and Bayesian posterior probabilities (PP) ≥ 0.95 were considered as significantly supported. The generated phylogenetic trees were visualized with FigTree v. 1.4.4 (Rambaut 2016).

## ﻿Results and discussion

The dataset includes a total of 104 sequences (42 ITS, 39 mtSSU, and 23 LSU, of which 8 ITS, 8 mtSSU, and 8 LSU are newly generated in this study) from 43 samples of 28 taxa (Suppl. material 1). The ML and BI trees showed similar topologies, so only the ML tree is provided here as Fig. 1. Compared with the dataset of Knudsen et al. (2023a), our phylogenetic analysis includes seven additional species (the three new species, *Trimmatothelopsiscoreana*, *T.ireneana*, *T.serpentinicola*, and *T.wendyana*).

Our phylogenetic tree revealed *Trimmatothelopsis* as a monophyletic genus. Three new lineages were recovered corresponding to three different species: *Trimmatothelopsisanthracina*, *T.shandongensis*, and *T.knudsenii*. The lineages of three new species were all strongly supported. The BS/PP of the lineages of *T.knudsenii* and *T.shandongensis* were 100/1 and the lineage of *T.anthracina* was 86/0.99.

In the phylogenetic tree, *Trimmatothelopsisanthracina* was sister to *T.coreana*, differing from *T.coreana* by its apothecia arising individually from the biofilm, carbonized apothecial disc, higher hymenium (200–250 μm vs. up to 190 μm) and thicker subhymenium (50–75 μm vs. up to 40 μm). *Trimmatothelopsisshandongensis* was closely related to *T.dispersa* and *T.versipellis*, *T.shandongensis* differs from other two species by its lower hymenium (120–140 μm), IKI+ red hymenial gel, and IKI+ blue ascus. *Trimmatothelopsisknudsenii* was recovered as an isolated lineage within the genus *Trimmatothelopsis*.

Three out of 21 species within the genus *Trimmatothelopsis* have no available gene sequences. Amongst these, *T.benedarensis* can be distinguished from our new species by well-developed rhizohyphae and smaller ascospores (3–4.5 × 1.5 μm) (Knudsen and Fox 2011; Knudsen et al. 2021c). *Trimmatothelopsismontana* differs from other species in this genus by its IKI+ blue-black hymenial gel (McCarthy 2008). *Trimmatothelopsiswirthii* can be distinguished from our new species by its large squamules up to 7 mm in width, which distinguish it from all other species in this genus (Roux 2021; Knudsen et al. 2023a). The available sequences for *T.americana* were limited to mtSSU, which proved insufficient for phylogenetic analysis. Therefore, it did not appear in our phylogenetic tree. In addition, our phylogenetic tree revealed a split between two specimens of *T.oreophila* which were identified as the same species by taxonomic features (Knudsen et al. 2021b). This incongruence was likely caused by incomplete molecular data: *T.californica* was represented by a three-locus dataset (ITS, LSU, and mtSSU), whereas *T.oreophila* lacked LSU sequence, limiting the analytical stability of its placement.

## ﻿Taxonomy

### ﻿ Trimmatothelopsis anthracina

Taxon classificationFungiAcarosporalesAcarosporaceae

J.X. Wang & L. Hu sp. nov.

90847976-0C7C-54BC-A113-EB71E85C5BB1

Fungal Names: FN 572513

Fig. 2

#### Diagnosis.

Similar to *Trimmatothelopsisversipellis* but differing in having IKI+ blue turning red hemiamyloid hymenial gel and in having the IKI+ blue ascus.

**Figure 2.**
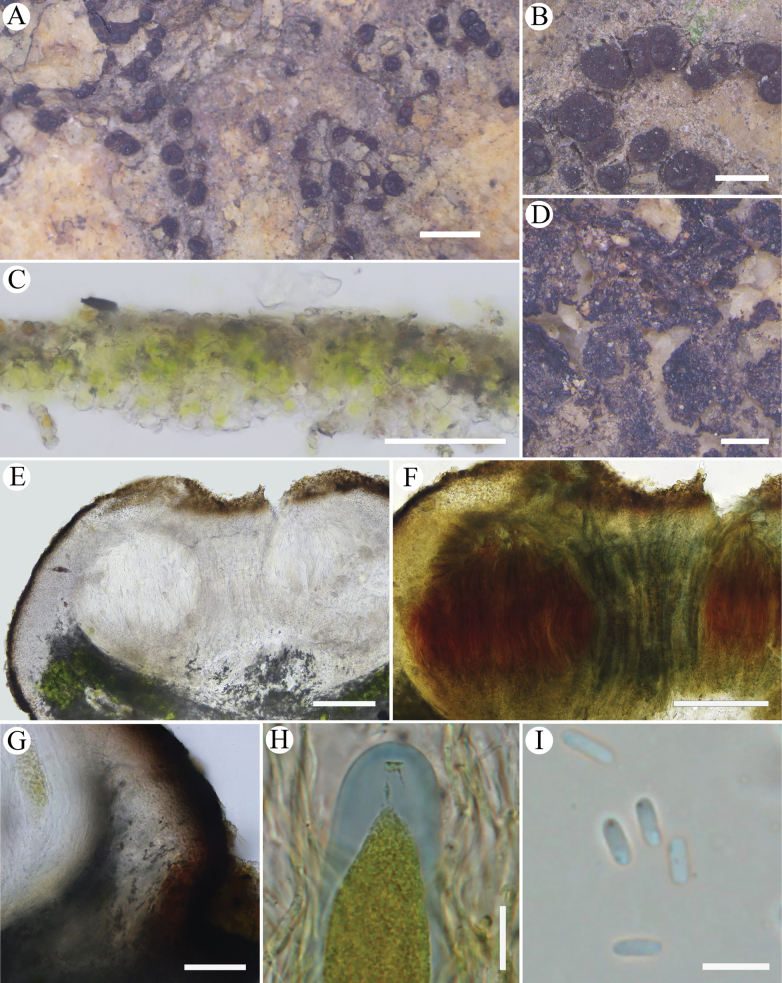
*Trimmatothelopsisanthracina*, (A–C, E–I. Holotype, SDNU 20242446; D. SDNU 20242244). A, B. Biofilm with apothecia; C. Section of biofilm; D. Dead biofilm; E. Section of apothecium; F. Hemiamyloid reaction of hymenium; G. Exciple; H. Ascus in IKI; I. Ascospores. Scale bars: 1mm (A); 500 μm (B); 200 μm (D); 100 μm (E, F); 50 μm (C, G); 10 μm (H); 5 μm (I).

#### Type.

China • Shandong Province: Yantai City, Muping District, Kunyu Mountain Nature Reserve, the steep slope near the road, 37°15'29.44"N, 121°43'12.02"E, alt. 304 m, on non-calcareous rock, 23 Nov. 2024, J.X. Wang et al. 20242446 (SDNU, holotype).

#### Etymology.

The epithet refers to the black carbonized apothecia when mature.

#### Description.

Thallus rare, of dispersed or contiguous irregular areoles, (0.15–)0.5–0.75 mm wide, sometimes indistinct, replicating by division, usually a biofilm composed of soil particles and algal cells at the immature apothecia base, ca. 30–50 μm thick, then dead when apothecia mature and easy separation from the substrate. Upper surface dull brown to black, rough, epruinose. Epicortex thin, ca. 5 μm thick. Cortex 25–40 μm thick, upper layer 10–15 μm thick, brown, lower layer hyaline. Algal layer continuous, uninterrupted, sometimes extending below apothecia, algal cells ca. 10 μm wide. Medulla obscured with crystals and gelatinization. Apothecia usually 1–3(–8) per areole, often with compound apothecia, 0.25–0.5 mm in diameter, brown to black, hemispherical, sometimes irregular, usually arising individually from the biofilm, initially punctiform, later dilated, sometimes arising individually from thallus, disc dull brown to black, usually carbonized, ca. 0.15 mm wide, usually circular, slightly concave or flat, sometimes with an umbo of the same color as the disc, (10–)12.5–15(–25) μm thick, rarely with slightly elevated apothecial crown. Out wall of apothecia (10–)12.5–15(–25) μm thick, carbonized, with inter thalline area sometimes absent, resulting in a space between the carbonized outer wall and the hymenium. Parathecium 40–50 μm thick, IKI-. Hymenium (200–)225–250 μm high, paraphyses 1.5–2 μm wide, apices expand, up to 4 μm wide, hymenial gel IKI+ blue turning red, hemiamyloid, if IKI too diluted with water on the slide, the reaction is IKI- pale yellow. Asci clavate, 110–140 × 15–25 μm, ascus stain IKI+ light blue tholus and space between the outer and inner wall of the ascus before ascospores fill the asci, ascospores several hundred per asci, narrowly ellipsoid, (4–)5–6(–7) × 1.5–2 μm. Subhymenium ca. 50–75 μm thick, IKI+, blue. Hypothecium obscured, ca. 10–20 μm thick, IKI-. Pycnidia not observed. Not producing secondary metabolites.

#### Habitat and distribution.

This new species is so far only known from Kunyu Mountain Nature Reserve, Shandong Province. It occurs on non-calcareous rock from the south-facing slopes located in the valley, at an elevation of 304 m.

#### Notes.

*Trimmatothelopsisanthracina* is similar to *T.americana*, *T.coreana*, *and T.versipellis* by its carbonized apothecial margin. *Trimmatothelopsisamericana* differs in its uncarbonized apothecial disc and small ascospores (3–5 × 0.5–1 μm) (Knudsen and Lendemer 2016). *Trimmatothelopsiscoreana* differs in its apothecia immersed in the thalline warts, no biofilm, uncarbonized apothecia disc, lower hymenium (up to 190 μm high), and lower subhymenium (up to 40 μm thick) (Kondratyuk et al. 2015). *Trimmatothelopsisversipellis* differs in its IKI+ blue euamyloid hymenial gel and IKI- ascus (Gueidan et al. 2014).

Before this study, *T.versipellis* was reported from Kunyu Mountain Nature Reserve where we found *T.anthracina* and *T.shandongensis*. However, the morphological re-examination of the sole specimen (Ren5389 SDNU) revealed significant discrepancies from the original description by Xiong et al. (2020). In Xiong’s description, this species has red-brown thallus, low hymenium (100–130 μm high), and IKI+ blue hymenial gel. However, during our re-examination of this specimen, it actually has brownish to dull brown thallus, globose apothecia, hymenium ca. 200 μm high, IKI+ blue turning red hymenial gel, IKI+ blue ascus, and ascospores 4.5–6.25 × 2.5–3 μm. Unfortunately, we could not sequence this specimen. Based on these characteristics, this specimen should not belong to *T.versipellis* which had carbon black thallus, IKI+ blue hymenial gel, IKI- ascus, and small ascospores 3–5 × 1–2 μm (Gueidan et al. 2014). However, this specimen shares many morphological and chemical characteristics with *T.anthracina*: dull brown thallus, high hymenium (ca. 200 μm), IKI+ blue turning red hymenial gel, and IKI+ blue ascus. Furthermore, both of them were collected along the Sancha River in the Kunyu Mountain Nature Reserve. In summary, we identified this specimen as *T.anthracina*.

#### Additional specimens examined.

China • Shandong Province: Yantai city, Muping District, Kunyu Mountain Nature Reserve, the steep slope near the road, 37°15'29.44"N, 121°43'12.02"E, alt. 304 m, on non-calcareous rock, 23 Nov. 2024, J.X. Wang et al. 20242211, 20242212, 20242224, 20242244, 20242447 (SDNU).

### ﻿ Trimmatothelopsis knudsenii

Taxon classificationFungiAcarosporalesAcarosporaceae

J.X. Wang & L. Hu sp. nov.

00EBD4A3-ADBC-5C01-B996-EE965E8B39BA

Fungal Names: FN 572514

Fig. 3

#### Diagnosis.

Similar to *Trimmatothelopsismontana* but differing in having wider thallus (up to 9 cm vs. up to 4 cm), in having completely immersed apothecia, and in having hymenial gel IKI+ blue turning red, hemiamyloid (vs. IKI+ blue-black amyloid).

**Figure 3.**
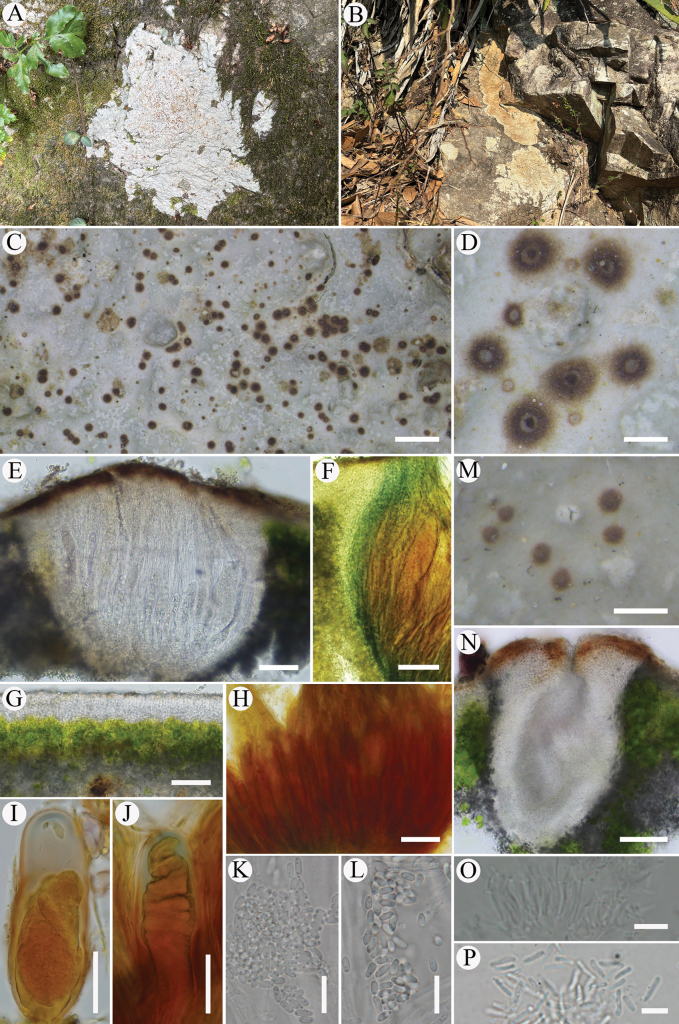
*Trimmatothelopsisknudsenii*, (A, C–P. Holotype, SDNU 20250049; B. SDNU 20250021). A, B. Habitat; C. Thallus with apothecia; D. Apothecia; E. Section of apothecium; F. IKI+ blue amyloid of parathecium; G. Section of cortex; H. Hemiamyloid reaction of hymenium; I, J. Ascus in IKI; K, L. Ascospores; M. Pycnidia; N. Section of pycnidia; O. Long narrow ampulliform conidiogenous cell producing conidia; P. Conidia. Scale bars: 1 mm (C); 200 μm (D, M); 50 μm (E–H, N); 20 μm (I, J); 10 μm (K–O); 5 μm (P).

#### Type.

China • Fujian Province: Quanzhou City, Fengze District, Qingyuan Mountain, Quankuguanpu, 24°56'49.14"N, 118°36'0.86"E, alt. 121 m, on non-calcareous rock, 1 Mar. 2025, J.X. Wang 20250049 (SDNU, holotype).

#### Etymology.

The species is named in honor of lichenologist and taxonomist Kerry Knudsen from the Czech Republic for his excellent revision of the genus *Trimmatothelopsis* and continuing work on the Acarosporaceae of California and southwestern North America, USA.

#### Description.

Hypothallus usually distinct, thick, grayish to greenish, irregular in outline. Thallus epilithic, continuous and rimose, to 9 cm wide, ca. 150 μm thick, pale greyish green, pale green, yellowish brown to brown, sparingly to richly rimose with cracks, somewhat pulpy when wetted, sometimes easily entirely peeling from rock. Epicortex absent. Cortex (5–)20–40 μm thick. Algal layer (25–)30–35(–75) μm thick, continuous, rarely interrupted by hyphal bundles, algal cells 5–9 μm wide. Medulla ca. 75 μm thick, obscured, sometimes interspersed with a few algal cells. Apothecia rather numerous, scattered, immersed, mostly solitary, sometimes with compound apothecia, disc up to 0.45 mm wide, usually level with the thallus, slightly convex or flat, rough or smooth, reddish brown or whitish with a brown circle at center, sometimes with slightly elevated brown apothecial crown, usually with a reddish brown ring around the base of mature apothecia. Parathecium 10–60 μm wide, merging with cortex, sometimes with an inter IKI+ blue, ca. 8–25 μm wide. Hymenium 150–230 μm high, paraphyses 1.5–2 μm wide, apices slightly widened in terminal brown gel cap, hymenial gel IKI+ blue turning red, hemiamyloid. Asci clavate, 90–100 × 20–30 μm, ascus stain IKI+ light blue tholus and space between the outer and inner wall of the ascus before ascospores fill the asci, ascospore several hundred per asci, ellipsoid to narrowly ellipsoid, 4–5(–6) × (1.5–)2–3 μm. Subhymenium obscure, 20–75 μm thick, IKI+ blue or sometimes light blue, with much oil drops. Hypothecium 20–25 μm thick, IKI-. Hymenium indistinct. Pycnidia immersed with dark ostioles, ca. 90 × 60 μm, with conidiogenous cells 22–28 × 1.5–2 μm, conidia rhabditiform, 5–6 × 1 μm. Not producing secondary metabolites.

#### Habitat and distribution.

This new species is so far only known from Qingyuan Mountain, growing on non-calcareous rock at 121–263 m from low hillsides. It commonly occurs in the type, living in full sun or in deeply shaded forest habitats.

#### Notes.

*Trimmatothelopsisknudsenii* has a rimose thallus which is an uncommon trait in the genus. Before this study, only one species was described with rimose thallus, *T.montana*. It can be distinguished from *T.knudsenii* by greenish black apothecia which look like perithecia and IKI+ blue-black hymenial gel (McCarthy 2008).

The morphological traits of this new species exhibit significant changes in response to habitat variation. Specimens from sun-exposed habitats usually have yellowish-brown to brown thallus with densely rimose surfaces. In contrast, specimens from shaded habitats usually have pale grayish-green to pale green thallus (rarely brown), with only sparse rimose cracking. Acarosporaceae usually occurs in fully sunny locations. When growing in shaded areas, they still receive sunlight for the majority of the day. In the family, only *T.montana* and *T.knudsenii* can tolerate deep shade.

#### Additional specimens examined.

China • Fujian Province: Quanzhou City, Fengze District, Qingyuan Mountain, Jianlongtai to Nanshanyan, 24°57'10.44"N, 118°35'47.04"E, alt. 263 m, on non-calcareous rock, 28 Feb. 2025, J.X. Wang 20250014, 20250015, 20250016, 20250017, 20250020, 20250021, 20250022, 20250023, 20250024, 20250025, 20250026, 20250027, 20250028, 20250029 (SDNU); • Quanzhou City, Fengze District, Qingyuan Mountain, Dafozi, 24°56'51.25"N, 118°36'2.36"E, alt. 175 m, on non-calcareous rock, 28 Feb. 2025, J.X. Wang 20250031, 20250032, 20250033, 20250034, 20250035, 20250036, 20250037, 20250038 (SDNU); • Quanzhou City, Fengze District, Qingyuan Mountain, Quankuguanpu, 24°56'49.14"N, 118°36'0.86"E, alt. 121 m, on non-calcareous rock, 1 Mar. 2025, J.X. Wang 20250039, 20250040, 20250041, 20250043, 20250044, 20250045, 20250046, 20250047, 20250048 (SDNU).

### ﻿ Trimmatothelopsis shandongensis

Taxon classificationFungiAcarosporalesAcarosporaceae

J.X. Wang & L. Hu sp. nov.

3C14B6BC-00F0-5D3D-BE56-136D12EA845E

Fungal Names: FN 572515

Fig. 4

#### Diagnosis.

Similar to *Trimmatothelopsismexicana* but differing in having dull brown thallus and apothecia, in having smaller apothecial disc (0.1 mm vs. 1–2 mm), and in having lower hymenium (120–140 μm vs. 200–220 μm).

**Figure 4.**
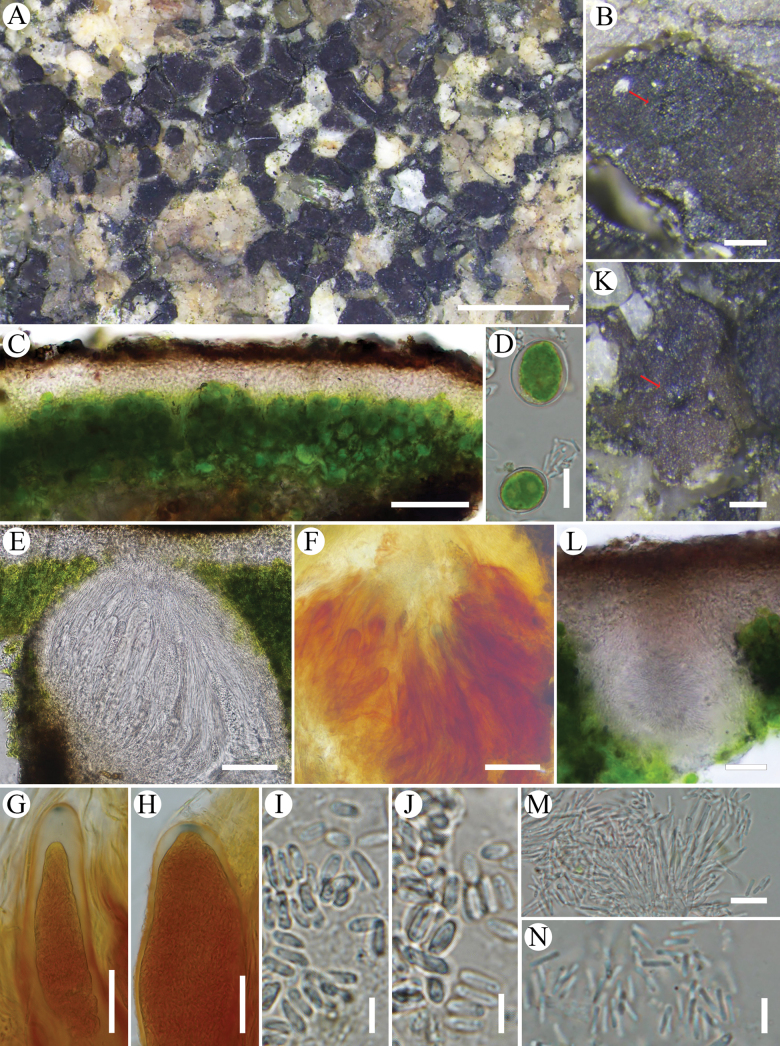
*Trimmatothelopsisshandongensis*, (holotype, SDNU 20242240a). A. Areolate thallus with apothecia; B. Apothecium; C. Section of cortex; D. Algal cells; E. Section of apothecium; F. Hemiamyloid reaction of hymenium; G, H. Ascus in IKI; I, J. Ascospores; K. Pycnidium; L. Section of pycnidium; M. Long narrow ampulliform conidiogenous cell producing conidia; N. Conidia. Scale bars: 1 mm (A); 50 μm (B, C, E, F, K); 20 μm (G, H, L); 10 μm (D, M); 5 μm (I, J, N).

#### Type.

China • Shandong Province: Yantai City, Muping District, Kunyu Mountain Nature Reserve, the steep slope near the road, 37°15'29.44"N, 121°43'12.02"E, alt. 304 m, on non-calcareous rock, 23 Nov. 2024, J.X. Wang et al. 20242240a (SDNU, holotype).

#### Etymology.

The specific epithet shandongensis refers to Shandong Province, where this species was found.

#### Description.

Thallus of dispersed or contiguous irregular areoles and subsquamules, 0.25–0.75 mm wide, replicating by division. Upper surface dull brown, usually with black undertone, rough, epruinose. Epicortex thin, ca. 5 μm thick. Cortex 30–50 μm thick, upper layer brown, ca. 8–12 μm thick, lower layer hyaline. Algal layer 50–75 μm thick, sometimes interrupted by hyphal bundles, algal cells mostly 8–15 μm wide. Medulla obscured with crystals and gelatinization, sometimes interspersed with a few algal cells. Apothecia rare, usually 1–2 per areole, immersed, disc up to 0.1 mm wide, concave, blackish brown, slightly darker than thallus, with slightly elevated apothecial crown, sometimes punctiform, epruinose. Parathecium indistinct, ca. 10–20 μm wide, merging with cortex, IKI-. Hymenium 120–140 μm high, paraphyses ca. 1.5 μm wide, apices unexpanded, lacking pigment caps, hymenial gel IKI+ red, hemiamyloid, if IKI too diluted with water on the slide the reaction is IKI- pale yellow. Asci clavate, 95–120 × 15–25 μm, ascus stain IKI+ light blue tholus and space between the outer and inner wall of the ascus before ascospores fill the asci, the blue area in upper layers of the tholus evanescent, ascospores several hundred per asci, narrowly ellipsoid, 5–7 × 2–3 μm. Subhymenium ca. 20 μm thick, hard to distinguish from hymenium, IKI-. Pycnidia immersed with dark ostioles, subglobose, 90–120 × 80–100 μm, with conidiogenous cells ca. 20 × 2 μm, conidia bacilliform, 4–6 × 1–1.5 μm. Not producing secondary metabolites.

#### Habitat and distribution.

This new species is only known from Kunyu Mountain Nature Reserve, Shandong Province, on non-calcareous rock at an elevation of 304 m. The holotype was collected on a steep sunny slope, growing with *T.anthracina*.

#### Notes.

*Trimmatothelopsisshandongensis* differs from other species in the genus *Trimmatothelopsis* by lower hymenium 120–140 μm high. The new species shares areolate to squamulose thallus and IKI+ red hymenial gel with *T.mexicana* and *T.oreophila*, but *T.mexicana* differs in having a pale brown thallus with stipe, brown apothecia, and the longest conidia (up to 13.9 μm long) in this genus (Knudsen et al. 2023a). *Trimmatothelopsisoreophila* differs from this new species in having apothecia with brownish red disc, IKI- ascus, and thicker subhymenium (30–50 μm vs. 20 μm) (Knudsen et al. 2021a). The dull brown surface of thallus was also found in *T.terricola*, but it differs in having rhizohyphae in root-like bundles and IKI- ascus (Knudsen 2007; Knudsen and Lendemer 2016). *Trimmatothelopsisshandongensis* also shares some features with *T.anthracina*: the blackish brown areolate thallus with completely immersed apothecia and IKI+ blue ascus, but *T.shandongensis* differs in its uncarbonized apothecia and IKI+ red hymenial gel.

#### Additional specimens examined.

China • Shandong Province: Yantai City, Muping District, Kunyu Mountain Nature Reserve, the steep slope near the road, 37°15'29.44"N, 121°43'12.02"E, alt. 304 m, on non-calcareous rock, 23 Nov. 2024, J.X. Wang et al. 20242240b (SDNU).

### ﻿Key to the species of *Trimmatothelopsis* in the world

**Table:** 

1	Ascus stain IKI+ blue	**2**
–	Ascus stain IKI	**11**
2	Thallus rimose	**3**
–	Thallus not rimose	**4**
3	Hymenial gel IKI+ blue black, Australia	***T.montana* (McCarthy 2008)**
–	Hymenial gel IKI+ blue turning red, China	***T.knudsenii* (this paper)**
4	With a Carbonized apothecial margin	**5**
–	Without a carbonized apothecial margin	**6**
5	Apothecial disc not carbonized, North America	***T.americana* (Knudsen et al. 2011 see microscopic pictures; Knudsen and Lendemer 2016)**
–	Apothecial disc carbonized, China	***T.anthracina* (this paper)**
6	On non-calcareous rock	**7**
–	On calcareous rock	**9**
7	Hymenium < 200 μm, China	***T.shandongensis* (this paper)**
–	Hymenium ≥ 200 μm, North America	**8**
8	Thallus areolate	***T.serpentinicola* (Knudsen et al. 2024)**
–	Thallus squamulose	***T.mexicana* (Knudsen et al. 2023a)**
9	Squamulose, France	***T.wirthii* (Roux 2021)**
–	Areolate	**10**
10	Areoles dispersed, France	***T.gordensis* (Navarro-Rosinés et al. 1999)**
–	Areoles contiguous, Chihuahuan Desert, New Mexico	***T.novomexicana* (Knudsen et al. 2023a)**
11	Ascospores globose 7–10(–12) μm, or broadly ellipsoid 7–9 × 5–7 μm, on base and calcareous rock, Asia, Europe, North America	***T.schorica* (Knudsen et al. 2011, 2021b, 2022**)
–	Ascospores ellipsoid, not globose or broadly ellipsoid, less than 7–9 × 5–7 μm	**12**
12	With a carbonized apothecial margin	**13**
–	Without a carbonized apothecial margin	**14**
13	Apothecial disc carbonized, coastal France	***T.versipellis* (Gueidan et al. 2014)**
–	Apothecial disc not carbonized, coastal Korea	***T.coreana* (Kondratyuk et al. 2015)**
14	On calcareous and non-calcareous rock, areoles brown, often with black margin, apothecial disc to 0.7 mm wide, North America	***T.dispersa* (Knudsen and Lendemer 2016)**
–	On non-calcareous rock or in soil crust	**15**
15	On non-calcareous rock	**16**
–	In soil crusts	**19**
16	Areolate	**17**
–	Squamulose	**18**
17	Areoles bullate to irregular, apothecia 1–6 per areole, North America	***T.californica* (Knudsen et al. 2023a)**
–	Areoles mostly plane, apothecia mostly one per areole, Korea	***T.ireneana* (Park et al. 2023)**
18	Apothecia solitary or up to 1–2 per squamule, conidia 3–5 × 1–1.5 μm, North America	***T.oreophila* (Knudsen et al. 2021b)**
–	Apothecia one to 20 per squamule, conidia 5–6 × 1 μm long, Korea	***T.wendyana* (Park et al. 2023)**
19	Rhizohyphae not in bundles, on compacted clay on sea cliffs, endemic to Ireland and U.K.	***T.benedarensis* (Knudsen et al. 2021b, c)**
–	Rhizohyphae in bundles	**20**
20	Rhizohyphal bundles, thick and root-like, 100–200 μm in diam., Greenland to U.K.	***T.rhizobola* (Knudsen and Lendemer 2016)**
–	Rhizohyphal bundles, not thick and root-like, 40–60 μm in diam., western North America	***T.terricola* (Knudsen and Lendemer 2016)**

## Supplementary Material

XML Treatment for Trimmatothelopsis anthracina

XML Treatment for Trimmatothelopsis knudsenii

XML Treatment for Trimmatothelopsis shandongensis

## ﻿Additional information

### Use of AI

No use of AI was reported.

### Author ORCIDs

Jiaxin Wang https://orcid.org/0009-0008-6376-4071

Fuhui Liang https://orcid.org/0009-0000-7210-0607

Zuntian Zhao https://orcid.org/0009-0007-2970-9231

Ling Hu https://orcid.org/0000-0002-9451-383X

### Data availability

All of the data that support the findings of this study are available in the main text or Supplementary Information.

## Supplementary materials

Supplementary material 1 A list of sampled specimens included in the molecular phylogeny Data type docx https://binary.pensoft.net/file/1393789This dataset is made available under the Open Database License (http://opendatacommons.org/licenses/odbl/1.0/). The Open Database License (ODbL) is a license agreement intended to allow users to freely share, modify, and use this Dataset while maintaining this same freedom for others, provided that the original source and author(s) are credited.Jiaxin Wang

Supplementary material 2 Phylogenetic tree constructed through BI analyses based on ITS, LSU, and mtSSU for Trimmatothelopsis Data type pdf Explanation note *Pycnorasorophora* was used as the outgroup. Only significant values (higher than 0.95 PP) are shown. https://binary.pensoft.net/file/1393790This dataset is made available under the Open Database License (http://opendatacommons.org/licenses/odbl/1.0/). The Open Database License (ODbL) is a license agreement intended to allow users to freely share, modify, and use this Dataset while maintaining this same freedom for others, provided that the original source and author(s) are credited.Jiaxin Wang
