# Supplementary material for: ﻿Three new taxa of lichen genus Trimmatothelopsis (Acarosporales, Acarosporaceae) from China
Source: MycoKeys. 2025 Aug 13;120:277–93. doi: 10.3897/mycokeys.120.158033 (PMC12368601; doi:10.3897/mycokeys.120.158033)
Supplement: Supplementary material 1 — A list of sampled specimens included in the molecular phylogeny [file mycokeys-120-277-s001.docx]

**Suppl. material 1.** Species, vouchers, localities, and GenBank accession numbers used for the phylogenetic analysis. Newly generated sequences are marked in bold. “–” indicates that there is no sequence available.

| **Species** | **Voucher** | **Locality** | **GenBank Accession Number** | | |
| --- | --- | --- | --- | --- | --- |
|  |  |  | **nrITS** | **mtSSU** | **nrLSU** |
| *Acarospora brattiae* | Dart 847.1 (SBBG) | U.S.A., California | ON303959 | ON303850 | ON303964 |
| *A. destructans* | Kocourkova 10557 (hb. K&K) | U.S.A., California | OM522311 | OM522315 | – |
| *A. privigna* | Kison 4432/3 (hb. K&K) | Germany, Saxony-Anhalt | OK142747 | OK032132 | ON303622 |
| *Myriospora bullata* | Schiefelbein 4763 (hb. K&K) | Germany, Sachsen-Anhalt | MZ262727 | MZ262739 | MZ262749 |
| *M. hassei* | Knudsen 707 (SBBG) | U.S.A, California | MW715698 | MW715737 | MW715726 |
| *Pleopsidium flavum* | Malicek (hb. Malicek) | Czech Republic, Prague | OK142757 | OK032142 | OP497841 |
| *Pycnora sorophora* | Hermansson 7903a (UPS L-111613) | Sweden, Härjedalen | FJ959357 | AY853338 | – |
| *Sarcogyne fallax* | Zaca 2347 (hb. K&K) | Portugal, Papagovas | MZ262722 | MZ262734 | MZ262744 |
| *S. hypophaea* | Knudsen 18343 (hb. K&K) | Italy, South Tyrol | OK142760 | OK032145 | ON303621 |
| *S. similis* | Dart 1332 (hb. K&K) | U.S.A., California | MW715720 | MW715741 | MW715730 |
| *Timdalia intricata* | Westberg (hb. K&K) | Sweden, Härjedalen | ON303957 | ON303848 | ON303962 |
| ***Trimmatothelopsis anthracina*** | **Wang 20242211 (SDNU)** | **Chian, Shandong** | **PV541751** | **PV541735** | **PV541743** |
| ***T. anthracina*** | **Wang 20242212 (SDNU)** | **Chian, Shandong** | **PV541752** | **PV541736** | **PV541744** |
| ***T. anthracina*** | **Wang 20240446 (SDNU)** | **Chian, Shandong** | **PV541753** | **PV541737** | **PV541745** |
| *T. californica* | Dart 577 (SBBG) | U.S.A., California | OP404922 | OP404919 | OP497839 |
| *T.coreana* | KH-L0007931 | South Korea | MT984218 | MW001364 | – |
| *T.coreana* | KH-L0007967 | South Korea | MT984215 | – | – |
| *T.coreana* | KH-L0012322 | South Korea | MT984230 | – | – |
| *T. dispersa* | Lendemer 7189 (SBBG) | U.S.A., Ohio | ON303960 | KX578714 | – |
| *T. gordensis* | CR22826 (MARSSJ) | France, Drôme | KM879337 | KM879331 | – |
| *T. gordensis* | CR25858 (MARSSJ) | France, Vaucluse | KM879338 | KM879332 | – |
| *T. ireneana* | KH-L0007356 | South Korea | MT984224 | MW001355 | – |
| *T. ireneana* | KH-L0007359 | South Korea | MT984225 | MW001356 | – |
| ***T. knudsenii*** | **Wang 20250036 (SDNU)** | **Chhina, Fujian** | **PV541756** | **PV541740** | **PV541748** |
| ***T. knudsenii*** | **Wang 20250049 (SDNU)** | **Chhina, Fujian** | **PV541757** | **PV541741** | **PV541749** |
| ***T. knudsenii*** | **Wang 20250041 (SDNU)** | **Chhina, Fujian** | **PV541758** | **PV541742** | **PV541750** |
| *T. mexicana* | Huereca AH-871 (PRM) | Mexico, Presa | OK142770 | OK032155 | OP497842 |
| *T. novomexicana* | Knudsen 19324 (SBBG) | U.S.A., New Mexico | OP162366 | OP404921 | OP216684 |
| *T. novomexicana* | Kocourkova 10875 (PRM) | U.S.A., New Mexico | OP404923 | OP404920 | OP497840 |
| *T. oreophila* | Knudsen 2410 (SBBG) | U.S.A., California | MK948458 | MK948477 | – |
| *T. oreophila* | Knudsen 2366 (SBBG) | U.S.A., California | MK948460 | MK948479 | – |
| *T. rhizobola* | Westberg 2994 (LD) | Sweden, Lule Lappmark | EU870640 | EU870692 | LN810868 |
| *T. rhizobola* | Westberg 3099 (LD) | Sweden, Lule Lappmark | EU870641 | EU870693 | – |
| *T. schorica* | Kocourkova 8980 (hb. K&K) | Czech Republic, Central Bohemia | ON303958 | – | – |
| *T. serpentinicola* | Carlberg02937B (CZU) | U.S.A., California | – | PP060616 | PP060617 |
| ***T. shandongensis*** | **Wang 20242240a (SDNU)** | **China, Shandong** | **PV541754** | **PV541738** | **PV541746** |
| ***T. shandongensis*** | **Wang 20242240b (SDNU)** | **China, Shandong** | **PV541755** | **PV541739** | **PV541747** |
| *T. terricola* | Knudsen 11216 & Sagar (S F256012) | U.S.A., California | LN810806 | LN810931 | – |
| *T. terricola* | Knudsen 11216 & Sagar (S F256013) | U.S.A., California | LN810807 | LN810932 | – |
| *T. versipellis* | CR25921 | France, Finistère | KM879336 | KM879327 | – |
| *T. versipellis* | CR25922 | France, Finistère | KM879335 | KM879328 | – |
| *T. wendyana* | KH-L0004196 | South Korea | MT984220 | MW001363 | – |
| *T. wendyana* | KH-L0007787 | South Korea | MT984221 | – | – |
