# Supplementary figures and images for: ﻿Three new taxa of lichen genus Trimmatothelopsis (Acarosporales, Acarosporaceae) from China
Source: MycoKeys. 2025 Aug 13;120:277–93. doi: 10.3897/mycokeys.120.158033 (PMC12368601; doi:10.3897/mycokeys.120.158033)

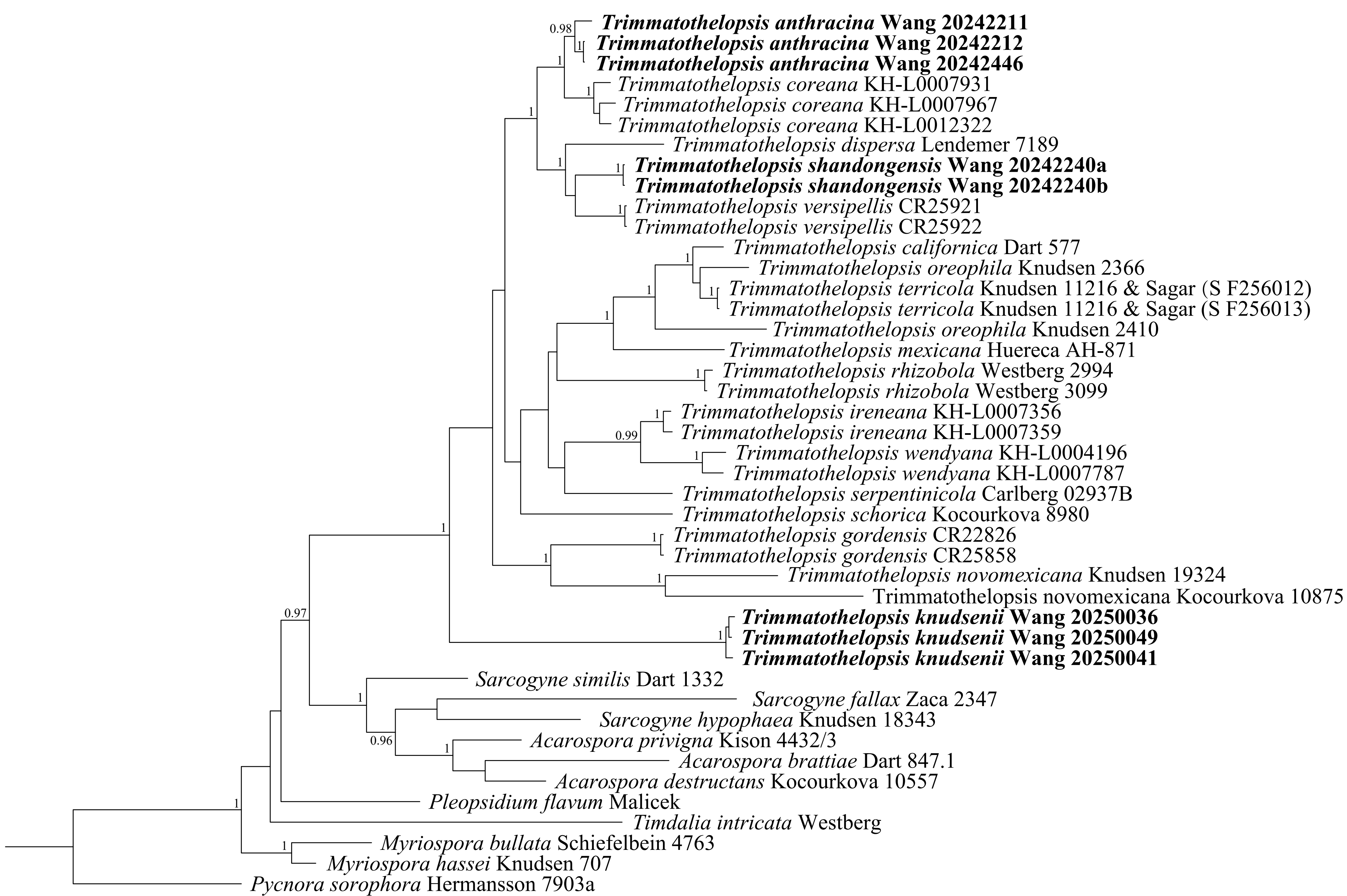

0.03

Supplement: Supplementary material 2 — Phylogenetic tree constructed through BI analyses based on ITS, LSU, and mtSSU for Trimmatothelopsis [file mycokeys-120-277-s002.pdf]
